# The role of TWEAK/Fn14 signaling in the MPTP-model of Parkinson’s disease

**DOI:** 10.1016/j.neuroscience.2016.01.034

**Published:** 2016-04-05

**Authors:** S. Mustafa, H.L. Martin, L. Burkly, A. Costa, M.L. Martins, M. Schwaninger, P. Teismann

**Affiliations:** a School of Medical Sciences, University of Aberdeen, Aberdeen, United Kingdom; b Department of Immunology, Biogen Idec, Inc., Cambridge, MA, United States; c Cell Death Regulation Laboratory, MRC Toxicology Unit, Leicester, United Kingdom; d Institute of Experimental and Clinical Pharmacology and Toxicology, University of Lübeck, Lübeck, Germany; e Department of Pharmacology, University of Heidelberg, Germany

**Keywords:** COX-2, cycoloxygenase-2, DOPAC, 3,4-dihydrophenylacetic acid, FN14, fibroblast growth factor-inducible 14, HPLC, high-performance liquid chromatography, HRP, horseradish peroxidase, MPTP, 1-methyl-4-phenyl-1,2,3,6-tetrahydropyridine, NF-κB, nuclear factor-kappaB, PBS, phosphate-buffered saline, PCA, perchloric acid, PD, Parkinson’s disease, SEM, standard error of the mean, SNpc, substantia nigra pars compacta, TBS, Tris-buffered saline, TH, tyrosine hydroxylase, TNF-α, tumor necrosis factor-alpha, TNFR1, TNF receptor 1, TWEAK, tumor necrosis factor like weak inducer of apoptosis, Parkinson’s disease, tumor-necrosis-factor-alpha, MPTP, TWEAK, Fn14

## Abstract

The tumor necrosis factor like weak inducer of apoptosis (TWEAK) and its receptor, fibroblast growth factor-inducible 14 (Fn14), mediate inflammation and neuronal apoptosis in cerebral edema, ischemic stroke and multiple sclerosis. The downstream effectors and pathways linked to TWEAK–Fn14 signaling are strongly implicated in the pathology of Parkinson’s disease (PD), thus indicating a putative role for TWEAK/Fn14 signaling in PD neurodegeneration. Using the 1-methyl-4-phenyl-1,2,3,6-tetrahydropyridine (MPTP) mouse model, we aimed to determine whether genetic ablation or pharmacologic mitigation of the TWEAK protein and its Fn14 receptor affected substantia nigra and striatum Parkinsonian pathology. Changes in endogenous TWEAK protein expression were also quantified in tissue from both MPTP-treated mice and PD human samples.

TWEAK protein expression was transiently increased in the striatal tissue but remained unaltered in substantia nigra tissue of MPTP-treated mice. There was also no change of TWEAK protein levels in the substantia nigra or the striatum of human PD patients as compared to matched control subjects. Mitigating the effects of endogenous TWEAK protein using neutralizing antibody did affect MPTP-mediated neurotoxicity in the substantia nigra using the sub-acute model of MPTP (30 mg/kg i.p. over five consecutive days). Neither TWEAK nor Fn14 genetic ablation led to attenuation of MPTP-toxicity in the acute model.

These findings suggest that TWEAK signaling might be an aspect of MPTP-mediated neuropathology and be involved in the overall neurodegenerative pathology of PD.

## Highlights

• We investigate the role of TWEAK and Fn14 in a model of Parkinson’s disease.
• Ablation of TWEAK or Fn14 had no effect on acute MPTP toxicity.
• TWEAK neutralizing antibody provided neuroprotection in the sub-acute MPTP-model.
• Suggestion of a possible role for TWEAK in Parkinson’s disease.

## Introduction

Parkinson’s disease (PD) is one of the main neurodegenerative disorders and is symptomatically characterized by debilitating movement impairments. The main underlying pathology of PD is the degeneration of dopaminergic neurons in the nigrostriatal pathway. Systemic administration of the neurotoxin 1-methyl-4-phenyl-1,2,3,6-tetrahydropyridine (MPTP) causes a similar nigrostriatal-selective dopaminergic cell loss in the mouse brain and therefore provides a suitable animal model to study PD neuropathology. Mechanisms such as microglial activation, oxidative stress and inflammatory cytokine release can mediate dopaminergic cell loss and their roles in PD neuropathology have been studied using the MPTP model (Litteljohn et al., 2010).

Members of the tumor necrosis factor (TNF) superfamily of inflammatory cytokines have been shown to be up-regulated in the substantia nigra (Boka et al., 1994) and the striatum (Mogi et al., 1994) of post-mortem brains of PD patients. Also, genetic deficiency of TNF-alpha signaling can confer neuroprotection against MPTP neurotoxicity in mice (Sriram et al., 2002, Ferger et al., 2004). One of the main apoptotic downstream effectors linked with TNF superfamily-related signaling in neuronal cells is the nuclear factor kappa-light-chain-enhancer of activated B cells (NFκB) signal transduction pathway (Aggarwal, 2003). NFκB nuclear translocation and activation mediates apoptotic effects via the tumor suppressor protein p53 (Ryan et al., 2000). In accordance with this concept, cultured dopaminergic neurons exposed to excitotoxic insult undergo apoptosis that is linked to NFκB-p53 signaling (de Erausquin et al., 2003). In the substantia nigra of PD patients, NFκB nuclear content is increased specifically in the remaining dopaminergic neurons indicating that the NFκB signal transduction pathway is relevant to PD neuropathology in human tissue (Hunot et al., 1997), as well as in the MPTP model (Dehmer et al., 2004). On the other hand we have to keep in mind that ablation of c-Rel leads to a late on-set of Parkinson-like features indicating a possible dual role – protective/promoting neurodegeneration of NF-κB (Baiguera et al., 2012).

More recently characterized members of the TNF superfamily include the TNF-like weak inducer of apoptosis (TWEAK) and its fibroblast growth factor-inducible 14 (Fn14) receptor (Chicheportiche et al., 1997, Wiley et al., 2001). TWEAK is a soluble cytokine widely expressed in neurons, astrocytes and macrophages and it acts as a ligand for the Fn14 receptor, the expression of which is up-regulated after tissue injury, including in injured hepatic, vascular and neuronal cells, and in tumors (Desplat-Jego et al., 2005, Winkles, 2008). TWEAK–Fn14 signaling has been linked to NFκB nuclear translocation and signal transduction (Brown et al., 2003, Tran et al., 2005). Furthermore, NFκB controls transcription of the gene for cycoloxygenase-2 (COX-2) which underlies PD neuropathology as demonstrated in the MPTP mouse model and observed in human PD tissue (Teismann et al., 2003). Thus, pro-inflammatory cytokines such as TWEAK can activate NFκB and its transcriptional control of downstream inflammatory mediators such as COX-2 and can therefore initiate and/or further exacerbate the neuroinflammatory and degenerative processes.

Neuronal death and neuroinflammatory processes have long been implicated in the pathogenesis of PD. Substantial evidence demonstrates that TWEAK–Fn14 signaling mediates neuroinflammatory processes and apoptotic cell death in *in vitro* and *in vivo* models of cerebral edema, ischemic stroke and multiple sclerosis (reviewed by Yepes (2007)). In models of pathological conditions such as ischemic stroke, chronic injury is associated with up-regulation of TWEAK and Fn14 expression (Potrovita et al., 2004, Inta et al., 2008). The neuronal cell death observed in these *in vivo* ischemia models, as well as in primary culture of cortical neurons undergoing oxygen–glucose deprivation, is associated with the observed increase in TWEAK and subsequent binding to Fn14 and activation of NF-κB expression (Potrovita et al., 2004, Polavarapu et al., 2005). Additionally it was shown, that the observed increase in the expression of monocyte chemoattractant protein-1 (MCP-1) and the recruitment of neutrophils after middle cerebral artery occlusion was absent in TWEAK^−/−^ and Fn14^−/−^ mice (Haile et al., 2010). Reactive microglia and astrocytes mediate inflammation processes that contribute to the neurodegenerative process in PD (Hirsch et al., 1999, Teismann and Schulz, 2004) and TWEAK has been shown to act on and mediate pro-inflammatory cytokine expression in astrocytes (Saas et al., 2000).

To date, little is known about the involvement of the TWEAK Fn-14 signaling complex in PD neurodegeneration. The aim of the present study is to determine the role of TWEAK–Fn14 in PD neuropathology as modeled using the MPTP neurotoxin in mice. We observed the effect of genetic ablation of TWEAK and Fn14 and neutralizing of TWEAK on MPTP-mediated neuropathologies such as dopaminergic cell loss in the substantia nigra, as well as dopamine depletion and fiber degeneration in the striatum.

## Experimental procedures

### Animals and treatment

Mice engineered with knockout of TWEAK or Fn14 (i.e. TWEAK^−/−^ and Fn14^−/−^) (Biogen Idec, Inc., Cambridge, MA, USA) (Jakubowski et al., 2005, Girgenrath et al., 2006), and wild-type littermates received an acute MPTP regime (4 × 18 mg/kg, intraperitoneal injections (i.p.), 2 h apart over one day). Mice were sacrificed 7 days after MPTP administration and their brain tissue was collected and processed for substantia nigra and striatal tyrosine hydroxylase (TH) immunohistochemistry and striatum monoamine high-performance liquid chromatography (HPLC) measurements.

For the neutralizing antibody experiments, adult male wild-type C57BL/6 mice weighing 20–25 g (Charles River Laboratories, Ormiston, UK) were treated with either an acute (4 × 18 mg/kg, i.p., 2 h apart) or sub-acute regimen of MPTP (5 × 30 mg/kg, i.p., over five consecutive days) and were injected i.p. with 200 μg anti-mouse TWEAK neutralizing antibody (Biogen Idec, Inc.) or isotype control antibody (Biogen Idec) 30 min prior to MPTP treatment (Potrovita et al., 2004). In the latter study the authors showed that the antibody crosses the blood–brain barrier. For striatum monoamine HPLC measurements and substantia nigra and striatal TH immunohistochemistry, mice undergoing the acute MPTP-regimen were killed 7 days after MPTP injection, mice undergoing the sub-acute MPTP-regimen 21 days after MPTP injection.

For endogenous TWEAK protein expression experiments, adult male wild-type C57BL/6 mice weighing 20–25 g (Charles River Laboratories, Ormiston, UK) were treated with an acute regime of MPTP (4 × 18 mg/kg, i.p., 2 h apart). Control mice received saline-vehicle. Sub-groups of mice were sacrificed at various time points following MPTP administration (0, 1, 2, 4, 7, 14 and 21 days). All procedures complied with the European Community Council Directive (2010/63/UE) and the Animals (Scientific Procedures) Act 1986 and were approved by the Home Office or the Regierungspräsidium Karlsruhe, Germany. Mice were housed in appropriately sized cages with access to food and water *ad libitum*, with a 12-hour light–dark cycle (lights on at 7 am).

### Human samples

Human nigral tissue was obtained from the UK Parkinson’s Disease Tissue Bank at Imperial College, London. Selected PD patients and controls were matched for age at death. Controls were (*n* = 5) 80.4 ± 2.66 years at death (mean ± SEM) and PD patients (*n* = 6) 79 ± 2.02 years with an average disease duration of 9.08 ± 1.20 years (mean ± SEM). The age at onset of disease seen was 69.83 ± 2 years. None of the PD patients had a family history of the disease. Samples were isolated in NP-40 buffer with protease inhibitors 1:20 (wt/vol) by hand homogenization. Extracts were centrifuged at 14,000 rpm (18,620×*g*; Mikro 200R) for 20 min at 4 °C and supernatants retained. All procedures were approved by the responsible ethics committee (North of Scotland Research Ethics Committees).

### Western blot analysis

The midbrain and striatum were dissected out from the mouse brain; total protein was isolated in ice-cold NP-40 buffer (20 mM Tris, 137 mM NaCl, 10% glycerol, 1% NP-40, 2 mM EDTA). The protein concentration was assessed using bicinchoninic acid (BCA) assay kit (Pierce, Rockford, IL, USA). 20 μg of protein (in Laemmli buffer and mercaptoethanol) was loaded onto a 12% SDS–polyacrylamide gel (SDS–PAGE) and transferred to a nitrocellulose membrane. After blocking with 5% milk powder in phosphate-buffered saline (PBS) containing 0.05% Tween-20 for 1 h the blots were incubated with the following primary antibodies: TWEAK (1:500, Cell Signalling) and β-actin (1:25,000, Sigma–Aldrich, UK) at 4 °C overnight, then rinsed with 0.05% PBS-Tween followed by an incubation at room temperature for 1 h with a horseradish peroxidase (HRP)-conjugated secondary antibody (anti-rabbit or anti-mouse 1:10,000, Amersham). After washing in PBS-Tween the blots were exposed to a homemade ECL solution (Luminol sodium salt in 0.1 mM Tris HCl and Para-hydroxycoumarin in DMSO) for 1–2 min and then viewed in the Alpha Imager and quantified using the Alpha imaging software.

Using the above protocol Western blot analysis was also performed on post mortem human brain samples.

### Immunohistochemistry studies

Mouse brains were dissected out and fixed in 4% paraformaldehyde (overnight at 4 °C) and then transferred to 30% (w/v) sucrose solution. The brains were then frozen by immersion in isopentane and stored at −80 °C. Coronal sections of frozen brains were cut (30 μm) in a cryostat. Ventral midbrain and striatal sections were collected in PBS with 0.01% sodium azide. The sections were washed with Tris-buffered saline (TBS) (three times for 5 min each), incubated with 10% methanol and 3% hydrogen peroxide, washed with TBS; incubated for 1 h in 5% normal goat serum followed by an incubation for 48 h with primary TH antibody (1:1000), rinsed with TBS (three times for 5 min each) and then incubated with biotinylated anti rabbit (1:200, Vector labs) antibody. After washes in PBS (three times for 5 min each) the sections were incubated for 1 h in HRP–avidin biotin complex (Vector), followed by washes in PBS (three times for 5 min each) and incubated for 15 min in diaminobenzidine (in 0.1 M Tris–HCl, pH = 7.6). All the sections were then mounted and dehydrated, counterstained with Nissl before viewing under a brightfield microscope. TH- and Nissl-positive cell counts in the substantia nigra pars compacta (SNpc) were quantified and estimated using the optical fractionator method (Stereoinvestigator, Microbrightfield Bioscience, Magdeburg, Germany) at 100× magnification. Striatal fiber staining was quantified by comparing the average optical density (AOD) of staining in the striatum with the surrounding cortical tissue, using Scion Image software.

### HPLC analysis of striatal monoamine levels

HPLC with electrochemical detection was used to measure striatal levels of dopamine, DOPAC and serotonin using a method that has been described (Sathe et al., 2012, Nuber et al., 2008). Briefly, after mice were killed, striata were dissected out and snap frozen directly on dry ice. Striata were then homogenized in 1% perchloric acid (PCA) (1:30 [i.e. tissue weight: PCA volume]) and centrifuged at 14,000*g* at 4 °C for 20 min. Following centrifugation, 20 μl of the supernatant sample was injected onto a C18 column (Dionex, Germering, Germany). The mobile phase consisted of 90% 50 mM sodium acetate, 35 mM citric acid, 105 mg/L octane sulfonic acid, 48 mg/L sodium EDTA solution, and 10% methanol at pH 4.3. Flow rate was 1 ml/min. Peaks were detected by an ESA Coulochem II electrochemical detector (ESA), and the detector potential was set at 700 mV. Data were collected and processed using the Chromeleon computer system (Dionex).

### Statistical analysis

All values expressed are mean ± the standard error of the mean (SEM). Differences between means were analyzed using Student’s *t*-test. Differences among means were analyzed using a one-way ANOVA. When ANOVA showed significant differences, pair-wise comparisons between means were assessed using the Newman–Keuls post hoc test. Null hypothesis was rejected at 0.05 levels. All analysis was performed using SPSS for windows and Graphpad Prism softwares.

## Results

### TWEAK protein expression in the acute MPTP model and human PD samples

**Fig. 1:**
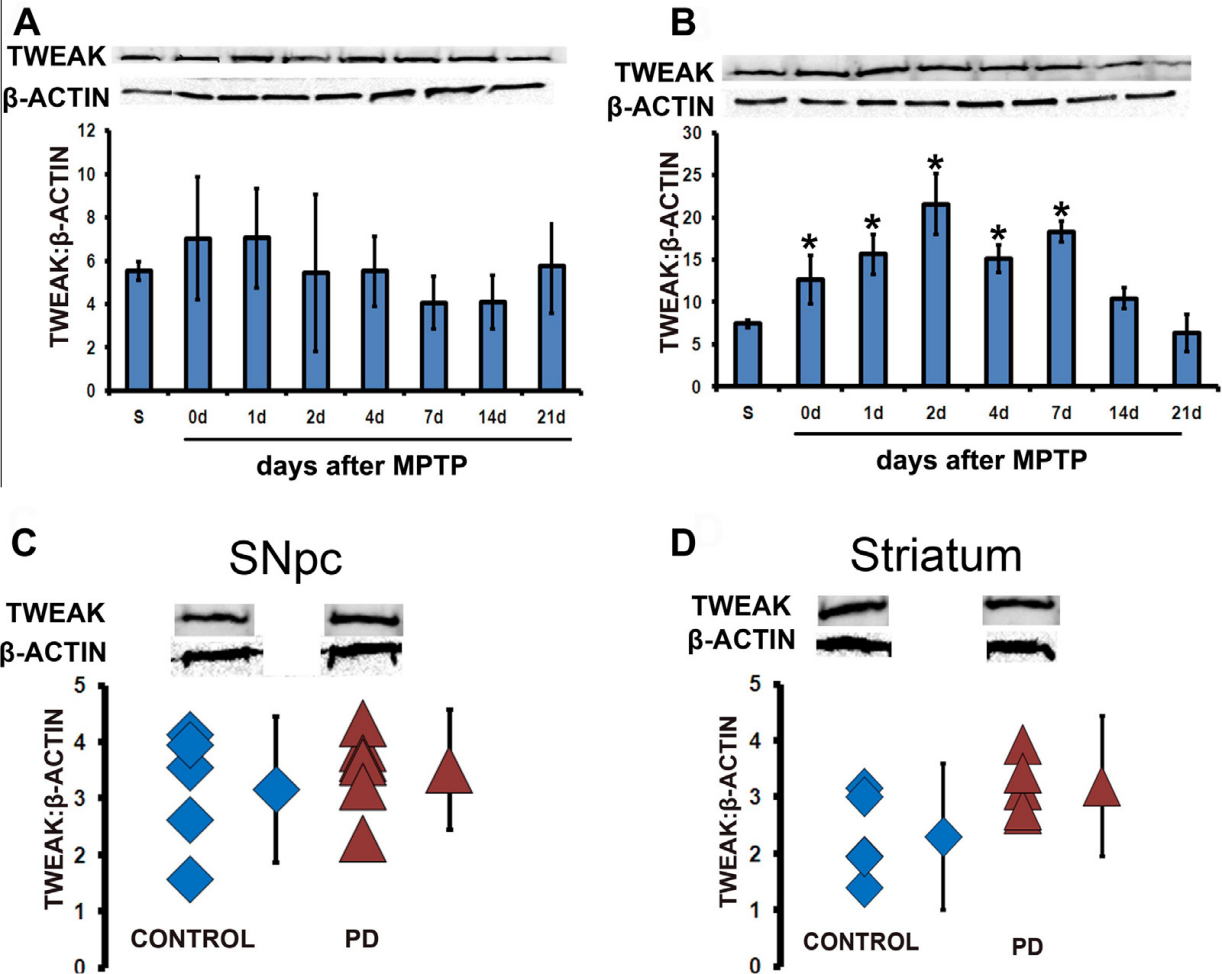
Protein expression of TWEAK in acute MPTP-treated mice and PD human samples. Western blot analysis of TWEAK protein levels normalized against β-actin levels in (A) SNpc and (B) striatal tissue extracted at various time points after acute MPTP treatment in mice. No change in protein levels of TWEAK was seen in the SNpc (A), however an increase in TWEAK expression is seen in the striatum (B) on days 2, 4, and 7 after MPTP treatment (*n* = 4–6; ^*^P < 0.05, compared with saline-treated animals [S], ANOVA with Newman–Keuls post-hoc test). Western blot analysis of TWEAK protein levels normalized against β-actin levels in (C) SNpc and (D) striatal tissue samples obtained from post-mortem human PD patients and control subjects. No change in protein expression was found in either the SNpc or striatum of post-mortem human PD samples.

To determine the expression pattern of TWEAK, we looked at the protein levels of TWEAK in the ventral midbrains and striata of saline and MPTP-treated mice at different time points. No change in nigral protein levels of TWEAK was seen in acute MPTP-treated mice when compared to saline at any of the various time points after MPTP treatment (*p* > 0.05, ANOVA with Newman–Keuls post-hoc, Fig.1A). However, an increase was seen in striatal levels of TWEAK protein on 2, 4 and 7 days after treatment with MPTP (^*^p = 0.03, compared to saline, ANOVA with Newman–Keuls post-hoc, Fig.1B).

The protein expression levels for TWEAK were also quantified in post-mortem human control and PD samples. No change was seen in TWEAK expression in either substantia nigra (Fig.1C) or striatal (Fig.1D) tissue of PD patients when compared to extracts from control human tissue.

### Genetic ablation of TWEAK and Fn14 does not alter acute MPTP toxicity

According to HPLC analysis, TWEAK and Fn14 ablation had no significant effect on levels of dopamine in the striatum compared to their wild-type counterparts (Table 1).

In the SNpc, the number of TH- and Nissl-positive dopaminergic neurons in acute MPTP-treated TWEAK^−/−^ or Fn14^−/−^ mice did not show any changes when compared to wild-type MPTP-treated mice (Table 1). Striatal TH-immunoreactivity appeared to be reduced in TWEAK^−/−^ mice in response to MPTP as compared to wild-type MPTP-treated mice, however, the effect was not significant (Table 1). Thus, genetic ablation of TWEAK^−/−^ or Fn14^−/−^ does not attenuate the effect of acute MPTP on SNpc dopaminergic cell counts and striatal dopaminergic fiber density.

### Neutralizing TWEAK mitigates effects of sub-acute MPTP on nigral dopaminergic neurons

**Fig. 2:**
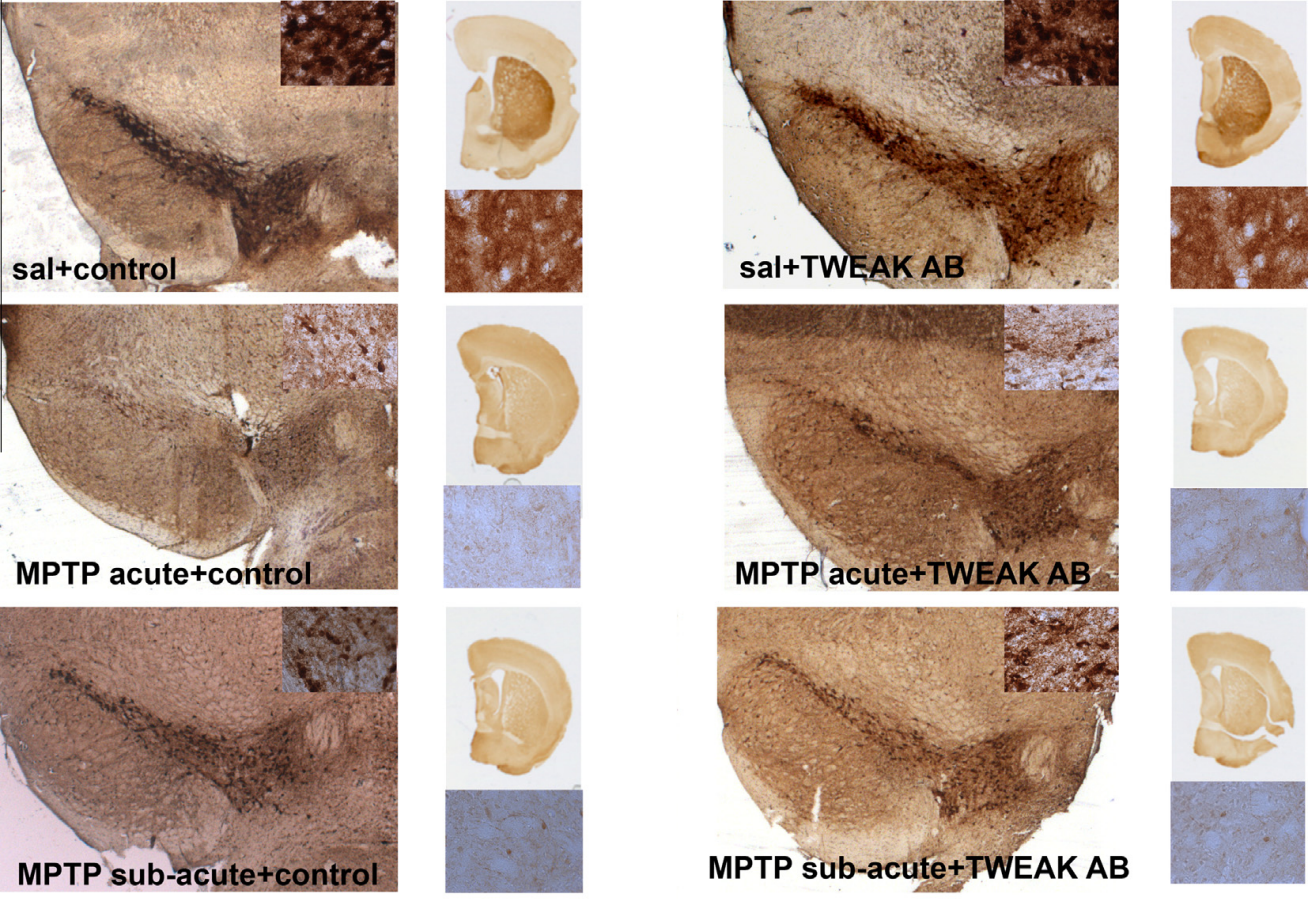
Effect of TWEAK neutralizing antibody on MPTP toxicity. Numbers of TH-positive neurons in the SNpc are comparable between saline-treated mice receiving TWEAK-neutralizing antibody and isotype control Ig (upper panel, Table 1). Three weeks after acute MPTP treatment, numbers of TH-positive neurons are not significantly different between mice receiving isotype control Ig or TWEAK neutralizing antibody (middle panel, Table 1); whereas after sub-acute MPTP-treatment mice initially receiving also TWEAK-neutralizing antibody treatment show higher numbers of TH-positive neurons, than do mice who received isotype control Ig (lower panel, Table 1). Striatal optical density was comparable between saline-treated mice receiving TWEAK-neutralizing antibody and isotype control Ig (upper panel, Table 1). Treatment with TWEAK neutralizing antibody had no effect on MPTP-induced loss of striatal optical density in the acute (middle panel, Table 1) or sub-acute (lower panel, Table 1) model. Pictures of SNpc at 2.5× magnification and inlay at 40× magnification. Pictures of striatum at 1× magnification, with inlay at 40× magnification.

Having established that TWEAK levels in the striatum are altered by MPTP treatment (Fig.1B), we assessed whether neutralization of TWEAK had an effect on MPTP-induced cell death. Neutralizing TWEAK did not lead to a change in SNpc cell counts of TH-positive dopaminergic neurons in mice dosed with acute MPTP at 18 mg/kg (Fig. 2 and Table 1) or at a lower MPTP dose of 16 mg/kg (data not shown) when compared to cell counts from mice that had received MPTP alone. Using the less severe sub-acute regimen significantly more dopaminergic neurons survived after MPTP-induced cell death in mice receiving the TWEAK antibody compared to controls treated with the isotype control Ig (Fig. 2 and Table 1).

In HPLC analysis we did observe higher striatal dopamine levels caused by neutralizing TWEAK antibody treatment combined with MPTP although not significant (Table 1).

## Discussion

Using both genetic knockout and neutralizing antibody methods, we aimed to determine whether TWEAK–Fn14 signaling is involved in MPTP-induced effects in the substantia nigra (SN) and striatum of mice. We also measured TWEAK protein expression in substantia nigra and striatal tissue from MPTP-treated mice and PD patients. Our results show that TWEAK-neutralizing antibody leads to attenuation of MPTP-induced dopaminergic cell death in the SNpc.

In both human and mouse substantia nigra tissue, no significant differences in TWEAK protein levels were observed between PD patients vs. healthy controls and MPTP-treated mice vs. saline-treated mice. However, in striatal tissue of MPTP-treated mice, there was increased TWEAK protein levels detected 2 days after treatment. This increase in striatal TWEAK protein was detected also on days 4 and 7 after MPTP-treatment. In post-mortem human striatal tissue from PD patients, we observed a trend of increased TWEAK protein in comparison to healthy controls, which if it were a statistically significant effect would have mimicked the transient striatal TWEAK expression seen in the mouse MPTP model. The expression levels of TWEAK and Fn14 proteins were also measured in human cortex samples obtained from healthy controls and PD patients with PINK and IPD mutations (data not shown). There was no difference in protein expression of either TWEAK or Fn14 between controls and the hereditary mutation samples suggesting that TWEAK and Fn14 expression changes are not associated with these hereditary forms of the disease and/or are not observed in the cortex.

It is interesting to observe at first view that TWEAK antibody is able to protect nigral dopaminergic neurons partially against MPTP-toxicity, whereas TWEAK as well as Fn14 ablation fails to provide any significant benefit. Different factors need to be kept in mind, which could have contributed to the observed differences. First of all, in the antibody study, a sub-acute regimen of MPTP administration was used. This model is linked closer to apoptotic induced cell death (Tatton and Kish, 1997), than the acute model, where cell death occurs mainly via necrosis (Jackson-Lewis et al., 1995). Another possibility could be that ablation of TWEAK or Fn14 leads to compensatory mechanisms, which then result in the observed effect, or anti-TWEAK resulted in Fc-mediated killing of TWEAK-expressing neuroinflammatory cells such as microglia.

Although we did not investigate glial activation in this study, it is interesting to note that TWEAK has been shown to be expressed in glial cells and has been reported to induce apoptosis by interacting with endogenous TNF-α and TNF receptor 1 (TNFR1) (Schneider et al., 1999). TNF-α expression in microglial cells has been shown in PD patients (Hunot et al., 1999). It has been demonstrated that TNFR1 ablation leads to neuroprotection in the MPTP-model of PD, suggesting the participation of TNF-α via TNFR1 in MPTP-induced cell death and in PD (Sriram et al., 2002, Ferger et al., 2004). As TNF-α and TNFR1 factors are mainly involved in apoptotic cell death, perhaps TWEAK as well as Fn14 ablation did not result in neuroprotection in the acute MPTP-model since cell death in the acute model mainly occurs via necrosis (Jackson-Lewis et al., 1995). Although a TWEAK antibody did provide partial neuroprotection in the sub-acute model, it was not a substantial protection, indicating, that the cell death processes in the MPTP-model are not mainly triggered via TWEAK. Although, TWEAK has been shown to interact with TNF-α, this seems not to be the main case in the MPTP-model. Also, we did not find a significant increase of TWEAK in the SNpc of PD patients.

Taking together our data suggest a minor role for TWEAK in PD. Still, when generating a neuroprotective therapy for the treatment of PD, one should keep in mind the potential role of TWEAK, and it might still prove to be a useful in a multi-target therapy, which in the end could be the way forward for the treatment of this debilitating disorder.

## Disclosures

L.B. is an employee and stockholder of Biogen Idec, Inc., which holds patents and pending patent applications in the United States and abroad on TWEAK-related molecules, including U.S. Patent No. 7129061, 7109298, 7087725, 7695934, 7566769, and 8048422.

## Figures and Tables

**Table 1:** Effects of TWEAK/Fn14 manipulation on MPTP-induced toxicity

	Dopamine (ng/mg wet tissue weight)	TH-positive neurons in the SNpc	Nissl cells in the SNpc	TH-positive fibers in the striatum (o.d. * 100)
*Saline*				
wt	11.367 ± 1.045	10790 ± 293	11370 ± 459	4.464 ± 0.619
FN14^−/−^	11.898 ± 1.140	9370 ± 138	10095 ± 1380	4.512 ± 0.249
TWEAK^−/−^	11.655 ± 1.095	9800 ± 637	10215 ± 465	4.630 ± 0.204
Scrambled antibody	12.246 ± 1.210	10475 ± 1863	10867 ± 845	4.850 ± 0.251
TWEAK antibody	11.624 ± 1.445	10380 ± 1894	9333 ± 1244	4.417 ± 0.590

*MPTP*				
wt	2.196 ± 0.266	4087 ± 556	4740 ± 392	0.0178 ± 0.097
FN14^−/−^	2.596 ± 0.597	3687 ± 493	4730 ± 400	0.0179 ± 0.090
TWEAK^−/−^	2.911 ± 0.439	3440 ± 810	4980 ± 539	0.0141 ± 0.071
Scrambled antibody acute MPTP	1.968 ± 0.449	3090 ± 530	4478 ± 172	0.030 ± 0.005
TWEAK antibody acute MPTP	1.534 ± 0.280	3464 ± 639	4387 ± 167	0.0233 ± 0.007
Scrambled antibody sub-acute MPTP	3.029 ± 0.355	5983 ± 269	7733 ± 473	0.030 ± 0.005
TWEAK antibody sub-acute MPTP	4.361 ± 0.753	7337 ± 454⁎	8410 ± 460	0.0233 ± 0.007

Values are mean ± SEM for six mice/group. Dopamine content was assessed by HPLC. Neuron and cell count were performed with stereology. Density of tyrosine hydroxylase (TH)-positive fibers was assessed by use of ScionImage Scion Corp., Frederick, Maryland, USA), and calculated using the formula: mean o.d. * 100.

⁎ *P* < 0.05 compared to numbers of TH-positive neurons in the SNpc in 1-methyl-4-phenyl-1,2,3,6-tetrahydropyridine (MPTP)-injected mice receiving scrambled antibody under the sub-acute regimen (Bonferroni post-hoc test).

## References

[b0005] Aggarwal B.B. (2003). Signalling pathways of the TNF superfamily: a double-edged sword. Nat Rev Immunol.

[b0010] Baiguera C., Alghisi M., Pinna A., Bellucci A., De Luca M.A., Frau L., Morelli M., Ingrassia R., Benarese M., Porrini V., Pellitteri M., Bertini G., Fabene P.F., Sigala S., Spillantini M.G., Liou H.C., Spano P.F., Pizzi M. (2012). Late-onset Parkinsonism in NFkappaB/c-Rel-deficient mice. Brain.

[b0015] Boka G., Anglade P., Wallach D., Javoy-Agid F., Agid Y., Hirsch E.C. (1994). Immunocytochemical analysis of tumor necrosis factor and its receptors in Parkinson’s disease. Neurosci Lett.

[b0020] Brown S.A., Richards C.M., Hanscom H.N., Feng S.L., Winkles J.A. (2003). The Fn14 cytoplasmic tail binds tumour-necrosis-factor-receptor-associated factors 1, 2, 3 and 5 and mediates nuclear factor-kappaB activation. Biochem J.

[b0025] Chicheportiche Y., Bourdon P.R., Xu H., Hsu Y.M., Scott H., Hession C., Garcia I., Browning J.L. (1997). TWEAK, a new secreted ligand in the tumor necrosis factor family that weakly induces apoptosis. J Biol Chem.

[b0030] de Erausquin G.A., Hyrc K., Dorsey D.A., Mamah D., Dokucu M., Masco D.H., Walton T., Dikranian K., Soriano M., Garcia Verdugo J.M., Goldberg M.P., Dugan L.L. (2003). Nuclear translocation of nuclear transcription factor-kappa B by alpha-amino-3-hydroxy-5-methyl-4-isoxazolepropionic acid receptors leads to transcription of p53 and cell death in dopaminergic neurons. Mol Pharmacol.

[b0035] Dehmer T., Heneka M.T., Sastre M., Dichgans J., Schulz J.B. (2004). Protection by pioglitazone in the MPTP model of Parkinson’s disease correlates with IkappaBalpha induction and block of NFkappaB and iNOS activation. J Neurochem.

[b0040] Desplat-Jego S., Creidy R., Varriale S., Allaire N., Luo Y., Bernard D., Hahm K., Burkly L., Boucraut J. (2005). Anti-TWEAK monoclonal antibodies reduce immune cell infiltration in the central nervous system and severity of experimental autoimmune encephalomyelitis. Clin Immunol.

[b0045] Ferger B., Leng A., Mura A., Hengerer B., Feldon J. (2004). Genetic ablation of tumor necrosis factor-alpha (TNF-alpha) and pharmacological inhibition of TNF-synthesis attenuates MPTP toxicity in mouse striatum. J Neurochem.

[b0050] Girgenrath M., Weng S., Kostek C.A., Browning B., Wang M., Brown S.A., Winkles J.A., Michaelson J.S., Allaire N., Schneider P., Scott M.L., Hsu Y.M., Yagita H., Flavell R.A., Miller J.B., Burkly L.C., Zheng T.S. (2006). TWEAK, via its receptor Fn14, is a novel regulator of mesenchymal progenitor cells and skeletal muscle regeneration. EMBO J.

[b0055] Haile W.B., Echeverry R., Wu J., Yepes M. (2010). The interaction between tumor necrosis factor-like weak inducer of apoptosis and its receptor fibroblast growth factor-inducible 14 promotes the recruitment of neutrophils into the ischemic brain. J Cereb Blood Flow Metab.

[b0060] Hirsch E.C., Hunot S., Faucheux B., Agid Y., Mizuno Y., Mochizuki H., Tatton W.G., Tatton N., Olanow W.C. (1999). Dopaminergic neurons degenerate by apoptosis in Parkinson’s disease. Mov Disord.

[b0065] Hunot S., Brugg B., Ricard D., Michel P.P., Muriel M.P., Ruberg M., Faucheux B.A., Agid Y., Hirsch E.C. (1997). Nuclear translocation of NF-kappaB is increased in dopaminergic neurons of patients with Parkinson disease. Proc Natl Acad Sci U S A.

[b0070] Hunot S., Dugas N., Faucheux B., Hartmann A., Tardieu M., Debre P., Agid Y., Dugas B., Hirsch E.C. (1999). FcεRII/CD23 is expressed in Parkinson’s disease and induces, in vitro, production of nitric oxide and tumor necrosis factor-alpha in glial cells. J Neurosci.

[b0075] Inta I., Frauenknecht K., Dorr H., Kohlhof P., Rabsilber T., Auffarth G.U., Burkly L., Mittelbronn M., Hahm K., Sommer C., Schwaninger M. (2008). Induction of the cytokine TWEAK and its receptor Fn14 in ischemic stroke. J Neurol Sci.

[b0080] Jackson-Lewis V., Jakowec M., Burke R.E., Przedborski S. (1995). Time course and morphology of dopaminergic neuronal death caused by the neurotoxin 1-methyl-4-phenyl-1,2,3,6-tetrahydropyridine. Neurodegeneration.

[b0085] Jakubowski A., Ambrose C., Parr M., Lincecum J.M., Wang M.Z., Zheng T.S., Browning B., Michaelson J.S., Baetscher M., Wang B., Bissell D.M., Burkly L.C. (2005). TWEAK induces liver progenitor cell proliferation. J Clin Invest.

[b0090] Litteljohn D., Mangano E., Clarke M., Bobyn J., Moloney K., Hayley S. (2010). Inflammatory mechanisms of neurodegeneration in toxin-based models of Parkinson’s disease. Parkinsons Dis.

[b0095] Mogi M., Harada M., Riederer P., Narabayashi H., Fujita K., Nagatsu T. (1994). Tumor necrosis factor-alpha (TNF-alpha) increases both in the brain and in the cerebrospinal fluid from Parkinsonian patients. Neurosci Lett.

[b0100] Nuber S., Petrasch-Parwez E., Winner B., Winkler J., von Horsten S., Schmidt T., Boy J., Kuhn M., Nguyen H.P., Teismann P., Schulz J.B., Neumann M., Pichler B.J., Reischl G., Holzmann C., Schmitt I., Bornemann A., Kuhn W., Zimmermann F., Servadio A., Riess O. (2008). Neurodegeneration and motor dysfunction in a conditional model of Parkinson’s disease. J Neurosci.

[b0105] Polavarapu R., Gongora M.C., Winkles J.A., Yepes M. (2005). Tumor necrosis factor-like weak inducer of apoptosis increases the permeability of the neurovascular unit through nuclear factor-kappa B pathway activation. J Neurosci.

[b0110] Potrovita I., Zhang W., Burkly L., Hahm K., Lincecum J., Wang M.Z., Maurer M.H., Rossner M., Schneider A., Schwaninger M. (2004). Tumor necrosis factor-like weak inducer of apoptosis-induced neurodegeneration. J Neurosci.

[b0115] Ryan K.M., Ernst M.K., Rice N.R., Vousden K.H. (2000). Role of NF-kappaB in p53-mediated programmed cell death. Nature.

[b0120] Saas P., Boucraut J., Walker P.R., Quiquerez A.L., Billot M., Desplat-Jego S., Chicheportiche Y., Dietrich P.Y. (2000). TWEAK stimulation of astrocytes and the proinflammatory consequences. Glia.

[b0125] Sathe K., Maetzler W., Lang J.D., Mounsey R.B., Fleckenstein C., Martin H.L., Schulte C., Mustafa S., Synofzik M., Vukovic Z., Itohara S., Berg D., Teismann P. (2012). S100B is increased in Parkinson’s disease and ablation protects against MPTP-induced toxicity through the RAGE and TNF-alpha pathway. Brain.

[b0130] Schneider P., Schwenzer R., Haas E., Muhlenbeck F., Schubert G., Scheurich P., Tschopp J., Wajant H. (1999). TWEAK can induce cell death via endogenous TNF and TNF receptor 1. Eur J Immunol.

[b0135] Sriram K., Matheson J.M., Benkovic S.A., Miller D.B., Luster M.I., O’Callaghan J.P. (2002). Mice deficient in TNF receptors are protected against dopaminergic neurotoxicity: implications for Parkinson’s disease. FASEB J.

[b0140] Tatton N.A., Kish S.J. (1997). *In situ* detection of apoptotic nuclei in the substantia nigra compacta of 1-methyl-4-phenyl-1,2,3,6-tetrahydropyridine-treated mice using terminal deoxynucleotidyl transferase labelling and acridine orange staining. Neuroscience.

[b0145] Teismann P., Schulz J.B. (2004). Cellular pathology of Parkinson’s disease: astrocytes, microglia and inflammation. Cell Tissue Res.

[b0150] Teismann P., Tieu K., Choi D.K., Wu D.C., Naini A., Hunot S., Vila M., Jackson-Lewis V., Przedborski S. (2003). Cyclooxygenase-2 is instrumental in Parkinson’s disease neurodegeneration. Proc Natl Acad Sci U S A.

[b0155] Tran N.L., McDonough W.S., Savitch B.A., Sawyer T.F., Winkles J.A., Berens M.E. (2005). The tumor necrosis factor-like weak inducer of apoptosis (TWEAK)-fibroblast growth factor-inducible 14 (Fn14) signaling system regulates glioma cell survival via NFkappaB pathway activation and BCL-XL/BCL-W expression. J Biol Chem.

[b0160] Wiley S.R., Cassiano L., Lofton T., Davis-Smith T., Winkles J.A., Lindner V., Liu H., Daniel T.O., Smith C.A., Fanslow W.C. (2001). A novel TNF receptor family member binds TWEAK and is implicated in angiogenesis. Immunity.

[b0165] Winkles J.A. (2008). The TWEAK-Fn14 cytokine–receptor axis: discovery, biology and therapeutic targeting. Nat Rev Drug Discov.

[b0170] Yepes M. (2007). Tweak and FN14 in central nervous system health and disease. Front Biosci.

